# The Use of Humoral Responses as a Marker of CMV Burden in HIV Patients on ART Requires Consideration of T-Cell Recovery and Persistent B-Cell Activation

**DOI:** 10.1155/2014/947432

**Published:** 2014-11-23

**Authors:** Samantha J. Brunt, Silvia Lee, Lloyd D'Orsogna, Christine Bundell, Sally Burrows, Patricia Price

**Affiliations:** ^1^School of Pathology and Laboratory Medicine, University of WA, Nedlands, WA 6009, Australia; ^2^Microbiology and Infectious Diseases, Royal Perth Hospital, Perth, WA 6000, Australia; ^3^Clinical Immunology and Immunogenetics, Royal Perth Hospital, Perth, WA 6000, Australia; ^4^Immunology & Immunopathology, PathWest Laboratory Medicine, Nedlands, WA 6009, Australia; ^5^Medicine and Pharmacology, University of WA, Nedlands, WA 6009, Australia

## Abstract

*Objectives.* Elevated humoral responses to cytomegalovirus (CMV) associate with increased risk of cardiovascular disease (CVD) in HIV patients on antiretroviral therapy (ART). To better understand the persistence of CMV humoral responses in relation to CVD, we determined trends in CMV antibody levels over the first 10 years on ART. *Design.* We describe longitudinal analyses of plasma from 13 HIV patients commencing ART with <210 CD4 T-cells/*µ*L and 27 controls. Antibodies reactive with CMV (fibroblast lysate, gB and IE-1 antigens), EBV-VCA, and HIVgp41 were quantitated. B-cell activation was assessed via total IgG and sBAFF. Inflammation was assessed via sTNF-RI and sCD14. *Results.* Amongst CMV seropositive HIV patients, levels of antibody reactive with CMV (*P* = 0.03) and EBV-VCA (*P* = 0.02) peaked after 1 year on ART. Levels of total IgG, sCD14, and sTNF-RI declined to approximate those in controls after 10 years, but sBAFF (*P* = 0.0002), EBV-VCA (*P* = 0.001), and CMV (*P* = 0.0004) antibodies remained elevated. A strong correlation between sBAFF and CMVgB antibody was seen at 10 years (*R* = 0.93, *P* = 0.0009) and verified in a second cohort. *Conclusions.* CMV antibody titres peak on ART and remain high. A correlation between CMV antibody and sBAFF suggests a role for HIV-induced B-cell pathology that may affect its use as a marker of CMV burden.

## 1. Introduction

Detection of cytomegalovirus (CMV) DNA in plasma associates with CMV end-organ disease and predicts mortality in untreated HIV patients [1, 2]. Antiretroviral therapy (ART) dramatically reduces the incidence of CMV-related conditions and presence of CMV DNA in plasma [3, 4]. However CMV infection leaves a distinct “footprint” on host immunity in the form of elevated humoral and T-cell responses to CMV. CMV seropositivity and/or T-cell responsiveness have been associated with accelerated T-cell immunosenescence and increased risk of cardiovascular disease (CVD) in HIV patients stable on ART [5, 6] and in the general aged population [7–9].

Untreated HIV infection causes immune activation and dysfunction affecting T-cells, monocytes, and B-cells. These changes are not completely attenuated by ART [10–12]. CD8 T-cell responses to CMV immediate-early protein 1 (IE-1) are elevated in HIV patients on ART and may reflect cyclical reactivation of CMV [13], but antibody responses to CMV IE-1 antigen have not been investigated. A role for CMV in ongoing immune dysfunction is evident from a study where treatment with the anti-CMV drug valganciclovir reduced CD8 T-cell activation in HIV patients on ART [14]. Hence strategies to reduce the “footprint” of CMV may reduce the incidence of CVD and onset of immunosenescence. However we must first understand how the CMV footprint is maintained in HIV patients responding to ART.

The interpretation of elevated levels of any CMV antibody is complicated by B-cell hyperactivation triggered by HIV disease. HIV affects B-cell phenotype, reducing expression of B-cell activating factor receptors (BAFF-R) that deliver survival and growth signals. Coupled with increased CD95 expression, this can promote apoptosis and consequent B-cell depletion [15]. B-cells that escape apoptosis in untreated HIV disease are hyperactive, manifesting as hypergammaglobulinemia and spontaneous autoantibody secretion [12, 16]. Levels of soluble (s) BAFF are elevated in untreated HIV infection [12, 17, 18]. In HIV-negative persons, B-cell numbers are inversely related to plasma levels of sBAFF [19] and levels of sBAFF are elevated in those with autoimmune disease [20]. Hence elevated sBAFF may be associated with autoantibody production in untreated HIV infection as both are features of B-cell pathology. This has not been addressed in patients stable on ART.

We hypothesized that levels of CMV antibody reflect the high burden of CMV established before ART and are maintained by perturbations to B-cell homeostasis caused by HIV and immune recovery on ART. We monitored changes in levels of CMV antibody over 10 years in HIV patients commencing ART with advanced disease, using in-house ELISAs that allowed extensive titration of samples to achieve accurate results in the high range. We explored the influence of B-cell activation using two plasma markers: total immunoglobulin G (IgG) and sBAFF. We also examined soluble tumour necrosis factor receptor 1 (sTNF-RI) to gauge chronic TNF*α* production and sCD14 to gauge macrophage activation. Although the study is small, the longitudinal design allowed evaluation of CD4 T-cell recovery, B-cell activation and inflammation as determinants of a humoral response to CMV at different times on ART amongst patients with low nadir CD4 T-cell counts. This is critical for understanding how humoral responses to CMV relate to disease outcomes such as CVD in HIV patients on ART.

## 2. Materials and Methods

### 2.1. Study Participants and Sample Collection

HIV patients were selected retrospectively from the HIV database of Royal Perth Hospital (Western Australia) on the basis of having achieved undetectable viral load (<50 copies per mL) within 6 months of commencing triple antiretroviral therapy (ART), baseline CD4 T-cell counts <210 cells/*μ*L, no evidence of HCV coinfection, and archived plasma samples available for analysis. Data were compared with Caucasian HIV-uninfected controls recruited in Western Australia. Blood was collected in lithium heparin tubes. Plasma samples were stored at −20 or −80°C and assayed simultaneously.

### 2.2. Immune Activation and Total IgG ELISAs

Commercial reagents were used to quantitate sTNF-RI, sCD14 (RnD Systems; Minneapolis, MN, USA) and sBAFF (Abcam; Cambridge, UK) in plasma. Patient plasma was titrated from 1 : 9 for sTNF-RI, 1 : 200 for sCD14, and 1 : 3 for sBAFF ELISAs. Binding of the peroxidase-conjugated secondary antibody was detected with a TMB substrate (tetramethylbenzene, Sigma-Aldrich; St Louis, MI, USA). Total IgG was quantified using plates coated with polyvalent goat anti-human IgG (Invitrogen, Carlsbad, CA, USA). Plasma samples were diluted from 1 : 100,000. Binding was detected using goat anti-human IgG conjugated HRP (Sigma-Aldrich) followed by TMB substrate, as described above. Reactions were stopped with 1 M H_2_SO_4_ and quantified spectrophotometrically at 450 nm (Bio-Rad microplate reader; Hercules, CA, USA). A 4-parameter logistic curve fit was generated from titrations of the standard, using SOFT max PRO version 5.4 software.

### 2.3. CMV, EBV, and HIV Antibody ELISAs

For HIVgp41 antibody ELISA, plates were coated with 100 ng/mL recombinant HIV-1 glycoprotein 41 (HIVgp41; NIH AIDS reagent program NIAID: Immunodiagnostics, Woburn, MA, USA) and plasma samples were titrated from 1 : 200. Bound IgG was detected as described above. Plasma of a HIV patient (not from this study) was titrated as a standard to give results in arbitrary units (AU/mL). IgG reactive with EBV viral capsid antigen (EBV-VCA) was detected using a commercial kit (DiaSorin, Vercelli, Italy). Plasma was diluted 1 : 1075 [21].

IgG reactive with CMV was detected using three antigens—CMV lysate, CMV glycoprotein B (gB), and CMV immediate-early protein 1 (IE-1). CMV lysate was prepared by sonication of human foreskin fibroblasts (HFF) infected with CMV strain AD169. Uninfected HFF were prepared in parallel to control for IgG reactive with HFF antigens. Patient plasmas were titrated from 1 : 1000. Anti-CMVgB and anti-CMV IE-1 antibodies were detected by coating plates with 50 ng/mL CMVgB (Chiron Diagnostics; Medfield, MA, USA) and 500 ng/mL CMV IE-1 (Miltenyi Biotech; Cologne, Germany). Plasma samples were diluted from 1 : 200 and bound IgG detected as described above. Plasma of a CMV seropositive healthy donor was used as a standard, providing results in AU/mL. A cut-off for CMV seropositivity was determined to be 1100 AU/mL of CMV lysate antibody. This was based on 11 persons confirmed to be CMV seronegative by routine serology performed at Royal Perth Hospital (Abbott Diagnostic Systems, Lake Forest, IL, USA).

### 2.4. Statistical Analysis

Analysis was performed using GraphPad Prism v5.04 software (La Jolla, CA, USA) and STATA 12 (StataCorp, College Station, TX, USA). Significance was set at alpha = 0.05. Mann Whitney and Fisher's exact tests were used to compare controls and patients. All correlations are nonparametric (Spearman) and used only values from a specified timepoint. Transformations were applied to approximate normal distributions [log (*ln*) for HIVgp41, CMVgB, and CMV lysate antibody and total IgG; square root (*sqrt*) for sTNF-RI and CD4 T-cell count; inverse (*inv*) for sBAFF]. Age, EBV-VCA antibody, and sCD14 did not require transformation. Random effects longitudinal regressions were used to investigate patterns and associations of variables over time. Nonlinear relationships were identified by comparison of fractional polynomial to linear models using Akaike Information Criteria (AIC), and best fit was determined. For CMV antibody outcomes (CMVgB and CMV lysate antigen), covariates were investigated individually (see Supplementary Table 1 in Supplementary Material available online at http://dx.doi.org/10.1155/2014/947432) and differences in the patterns of the association between covariates and CMV antibody over time were examined using interaction terms. The normality of each model residual was assessed visually by kernel density plots and statistically using Shapiro-Wilk tests (*P* > 0.05).

## 3. Results

### 3.1. Levels of CMV Antibody Increase in the First Year of ART and Remain Elevated for 10 Years

We quantitated CMV antibody in longitudinal plasma samples from 13 HIV patients and single samples from 27 healthy controls (25 males and 2 females, all Caucasian and aged 58 (50–74) years). Samples were available from patients at the commencement of ART (baseline, 13 patients) and after approximately 1 year (7–18 months, 12 patients), 2 years (19–25 months, 13 patients), 5–8 years (47–93 months, 7 patients), and 10 years on ART (102–149 months, 9 patients). Demographic and clinical information are provided in Tables 1(a) and 1(b). Eleven of 13 patients and 16 of 27 controls were CMV seropositive (Fisher's exact test, *P* = 0.15). Subsequent analyses were restricted to CMV seropositive HIV patients and controls.

**Figure 1:**
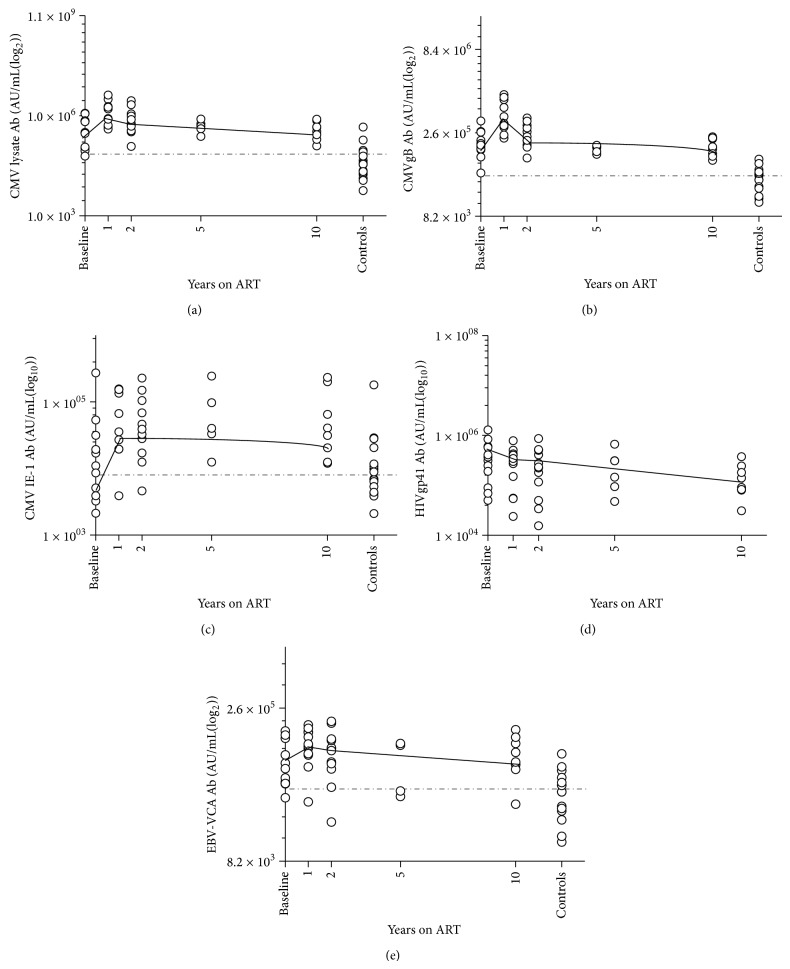
Levels of antibodies in HIV patients over 10 years of ART and in controls. Dotted lines indicated mean levels in controls. Solid lines indicate trends over time in HIV patients (based on position of mean). Levels of antibody reactive with CMV lysate (a), CMVgB (b), CMV IE-1 (c), and EBV-VCA (e) antigens increase in the first year of ART. Levels of HIVgp41 antibody decrease in a linear trend from commencement of ART.

The CMV seropositive HIV patients had levels of CMV antibody above those in control donors at all timepoints (baseline *P* = 0.0003, 1 year *P* < 0.0001, 10 years *P* = 0.004). Levels of antibody reactive with CMV increased from baseline levels in the first year of ART (*P* = 0.0009) but declined by the second year to approximate baseline levels (Figure 1(a)).

We further dissected trends of CMV antibody using the CMV antigens gB and IE-1, associated with neutralising and nonneutralising activity against CMV, respectively [22]. Levels of both antibodies were elevated above controls after 10 years of ART (CMVgB *P* = 0.0002; CMV IE-1, *P* = 0.011, Figures 1(b) and 1(c)). Similar to CMV lysate, antibody to CMVgB was elevated above levels in controls at baseline (*P* < 0.0001) and increased significantly in the first year of ART (*P* = 0.03), declining to approximate baseline levels in the second year. Antibody to CMV IE-1 was similar to controls at baseline (*P* = 0.74) but increased in the first year of ART (*P* = 0.003) and remained elevated after year 2 (*P* = 0.007).

### 3.2. Trends in CMV Antibody Were Similar to Those Detected with Epstein Barr Virus but Distinct from Responses to HIVgp41 Antigens

Levels of antibody to EBV VCA also increased from baseline levels in the first year of ART (*P* = 0.015) and were elevated above controls after 10 years (*P* = 0.001, Figure 1(e)). Antibodies reactive with HIV glycoprotein 41 (gp41) followed a linear trend of decline with continued ART distinct from the polynomial trend seen with CMV and EBV antibody. The difference was highlighted using multivariate linear regression modelling for (*ln*)CMVgB antibody, which yielded a significant interaction term for the covariate (*ln*)HIVgp41 (*P* = 0.007; Supplementary Table 1). This demonstrates a changing association between levels of HIVgp41 and CMV antibody with continued ART.

### 3.3. Levels of sBAFF Remained Elevated after 10 Years of ART but Inflammatory Markers Normalized More Rapidly

**Figure 2:**
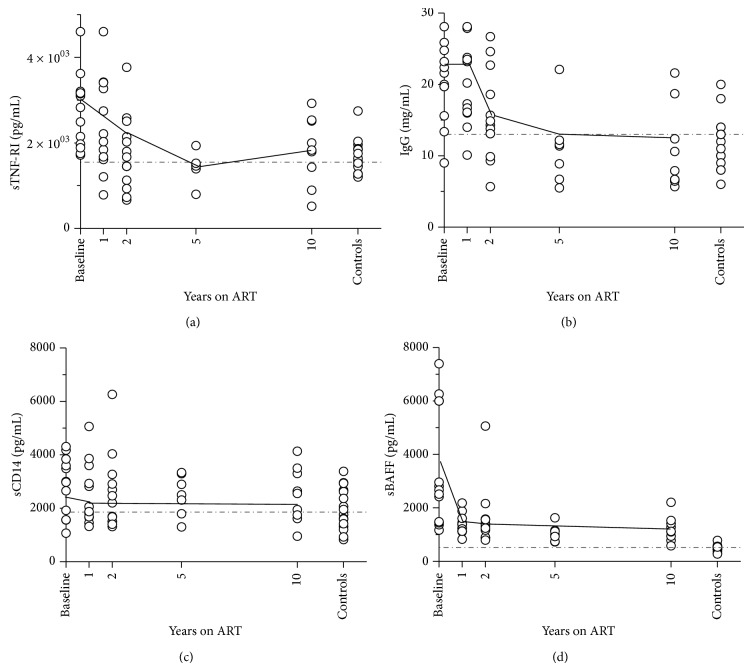
Levels of inflammatory markers in HIV patients over 10 years of ART and controls. Dotted lines indicated mean levels in controls. Solid lines indicate trends over time in HIV patients (based on position of mean). Levels of T-cell activation marker sTNF-RI (a), total immunoglobulin G (b), and macrophage activation marker sCD14 (c) decrease in HIV patients with continued ART, normalising to control levels after 10 years. Levels of B-cell activation marker sBAFF (d) decrease sharply in the first year of ART but remain elevated above controls after 10 years.

We asked if elevation in levels of CMV antibody reflected general B-cell activation and/or inflammatory markers. Levels of sTNF-RI (*P* = 0.0002), sCD14 (*P* = 0.02), sBAFF (*P* < 0.0001), and total IgG (*P* = 0.0001) were higher in untreated HIV patients than controls (Figure 2). After 10 years on ART, only levels of sBAFF remained elevated above levels in controls (*P* = 0.007; Figure 2(d)). We examined the associations of each marker with (*ln*)CMVgB antibody or (*ln*)CMV lysate antibody at all timepoints using linear regression of longitudinal models. Despite normalization in levels of (*sqrt*) sTNF-RI and (*ln*)total IgG, covariate associations were confirmed between CMV antibody and these variables, as well as CD4 T-cell count and (*inv*)sBAFF over 10 years. These associations were unchanging with time, with the exception of (*ln*)total IgG which generated a significant interaction term (*P* = 0.02; Supplementary Table 1; Figure 2(b)). Age and levels of sCD14 did not associate with CMV antibody over time.

### 3.4. Levels of CMV Antibody Correlated with Levels of sBAFF in HIV Patients on Long-Term ART

Levels of sBAFF at baseline correlated inversely with CD4 T-cell count (*R* = −0.61, *P* = 0.05) and HIVgp41 antibody (*R* = −0.61, *P* = 0.05) suggesting a link between sBAFF and the severity of HIV disease. There was no correlation between sBAFF and EBV-VCA antibody at any time (*R* < −0.08, *P* > 0.75), but levels of sBAFF and CMVgB antibody correlated in patients after 10 years of ART (*R* = 0.93, *P* = 0.0009). Weaker positive correlations were seen between sBAFF and CMV lysate antibody (*R* = 0.52, *P* = 0.11) and CMV IE-1 antibody (*R* = 0.83, *P* = 0.01) at 10 years. No correlations between sBAFF and CMV antibodies were seen in the control cohort (*R* < 0.11, *P* > 0.70 for all).

The correlation between sBAFF and CMVgB antibody was confirmed in another cross sectional HIV cohort from the same clinic [*n* = 20; 18 males, 2 females aged 62 (50–73) years] with nadir 42 (23–142) CD4 T-cells/*μ*L and current 691 (576–889) CD4 T-cells/*μ*L after 14 (13–15) years on ART. In this group of patients, levels of CMV antibody were also high [median (range) 3.2 × 10^6^ (4.1 × 10^5^ – 1.8 × 10^7^) AU/mL], and correlations were found between sBAFF and levels of antibody to CMVgB (*R* = 0.70, *P* = 0.002), CMV lysate (*R* = 0.72, *P* = 0.002), and CMV IE-1 (*R* = 0.54, *P* = 0.03). A multivariate linear regression confirmed that the association between (*ln*)CMV lysate antibody and (*inv*)sBAFF in these HIV patients was different from that seen in the control cohort (*n* = 27), as there was a significant interaction term (*P* = 0.04, Supplementary Table 2) for the integer “group” when modelling (*inv*)sBAFF and (*ln*)CMV antibody. A similar pattern was seen for (*ln*)CMV IE-1 antibody (*P* = 0.06). The data suggest that CMV antibody levels are linked to B-cell activation in CMV seropositive HIV patients after several years on ART, but not in healthy controls.

## 4. Discussion

In HIV patients commencing ART with <210 CD4 T-cells/*μ*L, we show that levels of CMV and EBV-VCA antibody increase during the first year of ART and remain elevated after 10 years. We further demonstrate levels of CMV antibody correlated with levels of sBAFF. We confirmed the association between CMV antibody and sBAFF in a second cohort of HIV patients on long- term ART and verified that it was not present in control donors.

We identified several factors contributing to the elevation in CMV and EBV reactive antibody after 1 year on ART. Here 10 of 11 CMV seropositive patients experienced an increase in CMV antibody titres within the first year of ART. There was no sample available for the 11th patient at 1 year. Two patients (7 and 11) had detectable CMV DNA prior to commencing ART, so antigen load prior to ART may drive production of CMV antibody when ART commences. CD4 T-cell recovery appears to boost CMV and EBV immunity, consistent with data from patients in our clinic showing that interferon-*γ* responses of T-cells stimulated with CMV antigens rise on ART [23].

However despite immediate recovery of CD4 T-cell function with commencement of ART, interferon-*γ* responses of T-cells stimulated with CMV antigens may decline after 3–5 years of ART [24, 25]. This likely reflects immunosenescence induced by HIV pathology [5]. Chronic persistent CMV infection promotes immunosenescence in older individuals independent of HIV [7–9], and HIV patients who are CMV seronegative have less phenotypic evidence of immunosenescence and greater immune resilience [26]. Associations between CMV humoral and T-cell responses to CMV and immunosenescence after >12 years on ART are described in a separate paper (submitted for publication).

Perturbation of B-cell homeostasis by HIV-infection must also be considered. ART reduces immune activation and restores immune cell populations but may not reverse HIV pathology. After 10 years of successful ART, antibodies to CMV and EBV remain at high levels, as does sBAFF (see Figures 1(a), 1(b), 1(c), 1(e), and 2(d)). Levels of CMV antibody and sBAFF correlate at this time, suggesting a role for unresolved B-cell activation in the maintenance of high CMV antibody titres. B-cell activation arises during untreated HIV infection and was evident here in the high levels of sBAFF and total IgG at baseline. ART increases numbers of circulating B-cells [19], which corresponds to a decrease in levels of sBAFF in the first year. However levels of sBAFF did not normalise. This agrees with independent studies showing that plasma markers of B-cell activation decrease but do not normalise in HIV patients after 2 years on ART [18]. Hence phenotypic changes to the B-cell population may also remain changed. Recognition of prevalent pathogens such as CMV and EBV by an increased number of B-cells with a readily matured phenotype could magnify the antibody response to CMV and EBV in the first year of ART and also explain the elevation in these responses up to 14 years later.

This study has limitations that arise from being retrospective in design, but the data raises some important considerations and draws strength over cross sectional studies from its longitudinal format. Future work will address the association of CMV antibody with increased risk of cardiovascular disease in ART patients, for which trends of CMV antibody established here will be an important consideration. Investigation into the phenotype of CMV-specific memory B-cells is also warranted.

## 5. Conclusions

The results establish that CMV antigen load before ART, CD4 T-cell recovery, and permanent changes to B-cell homeostasis require consideration when interpreting elevated humoral responses to CMV in long-term ART patients. This is critical when considering associations of the CMV footprint with health parameters such as immunosenescence and increased risk of cardiovascular disease, prominent issues for HIV patients on ART.

## Supplementary Material

Supplementary Tables 1 and 2 provide results of multivariate modelling and interactions described in the text. Table 1 provides results of longitudinal multivariate modelling for (ln)CMVgB antibody with time. Equation is polynomial, and was found to be similar for both (ln)CMVgB and (ln)CMV lysate antibodies. Levels of antibodies and inflammatory markers described in Figures 1 and 2 were tested as covariates in the model for (ln)CMVgB antibody over time. Results of associations for each covariate are reported in the Table, alongside interaction terms that were found to be significant. Supplementary Table 2 provides results of multivariate linear regression modelling for levels of (ln)CMV lysate, CMVgB or CMV IE-1 antibody with (inv)sBAFF for HIV patients and controls, demonstrating a difference in the association of CMVgB antibody and sBAFF in HIV patients compared to controls.

## Figures and Tables

**Table 1a:** (a) Characteristics of HIV patient cohort

	Sex	Age	Ethnicity	CD4 T-cells/*μ*L		CMV DNA	CMV IgG	Comments
				Nadir	1 year			
HIV cohort								

1	M	40	Caucasian	42	112	ND	Pos.	CCR5 delta 32 deletion
2	F	28	Asian	8	352	ND	Neg.	
3	F	43	Aboriginal	90	378	N/A	Pos.	IRD-HSV, 1 month after ART
4	M	42	Caucasian	33	28	ND	Pos.	IRD-CMV, 7 months after ART
5	M	47	Caucasian	110	169	N/A	Pos.	
6	M	43	Caucasian	4	465	ND	Neg.	
7	M	56	Caucasian	9	176	D	Pos.	Oesophageal tumour before ART
8	M	45	Caucasian	70	105	ND	Pos.	Syphilis, 8 years after ART
9	M	48	Asian	18	189	ND	Pos.	IRD-uveitis, 1 month after ART
10	F	60	Caucasian	56	220	D	Pos.	HIV encephalitis, before ART
11	M	39	Caucasian	208	240	D	Pos.	CMV pneumonitis, before ART
12	M	67	Asian	14	250	ND	Pos.	Died age 76
13	F	60	Caucasian	4	108	N/A	Pos.	Died age 65, breast cancer

**Table 1b:** (b) Comparison of CMV seropositive HIV and control cohorts

*n*	Male/female	Age median (range)	Ethnicity	CMV lysate antibody AU/mL median (range)	CMVgB antibody AU/mL median (range)	CMV IE-1 antibody median (range)
HIV^+^CMV^+^ persons at 10 years after ART						

11	3/8	57 (49–77)	1 Aboriginal 2 Asian 10 Caucasian	4.1 × 10^6^ (1.3 × 10^6^–8.3 × 10^6^)	1.4 × 10^6^ (8.3 × 10^5^–2.2 × 10^6^)	3.6 × 10^5^ (1.1 × 10^4^–2.3 × 10^6^)

CMV^+^ Controls						

16	14/2	58 (50–74)	16 Caucasian	3.6 × 10^5^ (5.9 × 10^4^–4.8 × 10^6^)	4.5 × 10^5^ (1.4 × 10^4^–8.8 × 10^5^)	7.9 × 10^4^ (2.1 × 10^4^–1.8 × 10^6^)
