# Supplementary material for: The Use of Humoral Responses as a Marker of CMV Burden in HIV Patients on ART Requires Consideration of T-Cell Recovery and Persistent B-Cell Activation
Source: Dis Markers. 2014 Nov 23;2014:947432. doi: 10.1155/2014/947432 (PMC4259131; doi:10.1155/2014/947432)
Supplement: Supplementary file 1 — Supplementary Tables 1 and 2 provide results of multivariate modelling and interactions described in the text. Table 1 provides results of longitudinal multivariate modelling for (ln)CMVgB antibody with time. Equation is polynomial, and was found to be similar for both (ln)CMVgB and (ln)CMV lysate antibodies. Levels of antibodies and inflammatory markers described in Figures 1 and 2 were tested as covariates in the model for (ln)CMVgB antibody over time. Results of associations for each covariate are reported in the Table, alongside interaction terms that were found to be significant. Supplementary Table 2 provides results of multivariate linear regression modelling for levels of (ln)CMV lysate, CMVgB or CMV IE-1 antibody with (inv)sBAFF for HIV patients and controls, demonstrating a difference in the association of CMVgB antibody and sBAFF in HIV patients compared to controls. [file 947432.f1.pdf]

Interaction models for (ln) CMVgB antibody over time

Variable	Time term 1<br>W<br>Coefficient<br>(95% CI)<br>p-value	Time term 2<br>Z<br>Coefficient<br>(95% CI)<br>p-value	Covariate<br>S<br>Coefficient<br>(95% CI)<br>p-value	Interaction Time term 1<br>P<br>Coefficient<br>(95% CI)<br>p-value	Interaction Time term 2<br>Q<br>Coefficient<br>(95% CI)<br>p-value	Constant<br>(95% CI)<br>p-value
none	0.47<br>(0.07, 0.88)<br>**p=0.02**	6.64<br>(4.32, 8.96)<br>**p<0.0001**				11.6<br>(11.3,12.0)<br>**p<0.0001**
(*ln*) HIVgp41 Ab	1.28<br>(0.50, 2.07)<br>p=**0.002**	0.52<br>(-0.19, 0.84)<br>p=**0.002**	-0.02<br>(0.26,0.22)<br>p=0.88	-0.09<br>(-0.16, -0.03)<br>**p=0.006**	-0.04<br>(-0.06, -0.01)<br>**p=0.007**	12.5<br>(9.6,15.3)<br>**p<0.0001**
(*ln*) IgG	-0.28<br>(-0.64, 0.07)<br>p=0.11	0.11<br>(-0.26, 0.11)<br>p=0.18	0.56<br>(0.08,1.03)<br>**p=0.02**	0.14<br>(0.01, 0.27)<br>**p=0.02**	0.05<br>(0.002, 0.11)<br>**p=0.042**	10.7<br>(9.4,11.9)<br>**p<0.0001**
Age	0.15<br>(0.09, 0.21)<br>**p<0.0001**	0.06<br>(0.04, 0.09)<br>**p<0.0001**	-0.01<br>(-0.03, 0.02)<br>p=0.51			12.3<br>(12,12.5)<br>**p<0.0001**
(*sqrt*) CD4 T-cell	0.23<br>(0.15,0.31)<br>**p<0.0001**	0.094<br>(0.62,0.13)<br>**p<0.0001**	0.018<br>(0.015, 0.088)<br>**p=0.006**			12.10<br>(11.9,12.3)<br>**p<0.0001**
sCD14	0.15<br>(0.10,0.21)<br>**p<0.0001**	0.06<br>(0.04,0.09)<br>**p<0.0001**	$8.2x10^{-6}$<br>($-1x10^{-3}$, $2x10^{3}$)<br>p=0.93			12.2<br>(12.0,12.5)<br>**p<0.0001**
(*ln*) IE-1 Ab	0.15<br>(0.10,0.19)<br>**p<0.0001**	0.06<br>(0.04,0.08)<br>**p<0.0001**	0.24<br>(0.07, 0.41)<br>**p=0.005**			12.2<br>(11.9,12.5)<br>**p<0.0001**
(*sqrt*) sTNF-RI	0.14<br>(0.09,0.20)<br>**p<0.0001**	0.06<br>(0.38,0.08)<br>**p<0.0001**	0.02<br>(0.002,0.040)<br>**p=0.03**			12.3<br>(12.0,12.5)<br>**p<0.0001**
(*inv*) sBAFF	0.15<br>(0.099,0.20)<br>**p<0.0001**	0.06<br>(0.04,0.08)<br>**p<0.0001**	-0.0001<br>($-2x10^{-3}$, $3x10^{-4}$)<br>**p=0.006**			12.2<br>(11.9,12.4)<br>**p<0.0001**
EBV-VCA Ab	0.16<br>(0.10,0.23)<br>**p<0.0001**	0.06<br>(0.04,0.09)<br>**p<0.0001**	$-6.3x10^{-7}$<br>($-4.7x10^{-6}$, $3.5x10^{-6}$)<br>p=0.76			12.2<br>(12.0,12.5)<br>**p<0.0001**

Supplementary Table 1. Results of model fitting for CMV antibody. This table reports model parameters to support results of the main text. Associations of each immune parameter with CMVgB antibody, as well as changes in these associations with continued ART were investigated using the regression model for (*ln*)CMVgB antibody over time (*equation 1*). (*ln*)HIVgp41 antibody and (*ln*)total IgG were found to have a significant change in their association with (*ln*)CMVgB antibody over time (indicated by Interaction time terms P and Q in table). (*sqrt*)CD4 T-cell counts, (*ln*)CMV IE-1 antibody, (*sqrt*)sTNF-RI and (*inv*)sBAFF were found to associate with (*ln*)CMVgB antibody, but no variation was found in these associations with time (P and Q interaction terms p>0.05, and hence omitted). Age, sCD14 and EBV-VCA antibody were found to have no association with (*ln*)CMVgB antibody over time.

Equation 1: fractional polynomial describing (*ln*)CMVgB antibody over time with continued ART. Z, W are time term coefficients. S is the coefficient for the covariate in the model, P and Q are coefficients for interaction terms.

$$\boldsymbol{(ln)CMvgB\ antibody}$$

$$= Z\left[\left(\frac{Timepoint+1}{10}\right)^{-2} - 6.11\right] + W\left[\left(\frac{Timepoint+1}{10}\right)^{-2} \times ln\left(\frac{Timepoint+1}{10}\right) + 5.534\right]$$

$$+ S[Variable] + P\left[\left(\frac{Timepoint+1}{10}\right)^{-2} - 6.11\right] * [Variable]$$

$$+ Q\left[\left(\frac{Timepoint+1}{10}\right)^{-2} \times ln\left(\frac{Timepoint+1}{10}\right) + 5.534\right] * [Variable] \ + constant$$

Outcome	A. Group Coefficient (95% CI) p-value	B. (*inv*)sBAFF Coefficient (95% CI) p-value	C. (inv)sBAFF Coefficient (95% CI) p-value	D. Interaction term Coefficient (95% CI) p-value	Constant Coefficient (95% CI) p-value
(*ln*)CMV lysate antibody	3.2<br>(1.5,4.9)<br>**0.001**	-78<br>(-664,508)<br>0.79	-1064<br>(-1787, -341)<br>**0.006**	-985<br>(1916,55)<br>**0.04**	9.9<br>(8.6,11.17)<br>0.0001
(*ln*) CMVgB antibody	2.6<br>(-0.9,4.3)<br>**0.004**	258<br>(-327,843)<br>0.40	-495<br>(-1216,226)<br>0.17	-753<br>(-1682,175)<br>0.11	9.9<br>(8.6,11.20)<br><0.0001
(*ln*)CMV IE-1 antibody	3.8<br>(1.2,6.4)<br>**0.006**	232<br>(-675,1139)<br>0.60	-1157<br>(-2275,-38)<br>**0.04**	-1388<br>(-2830,52)<br>0.06	8.7<br>(6.7,10.7)<br><0.0001

Supplementary Table 2. Coefficients and confidence intervals for multivariate modelling, with antibodies reactive with each of the 3 CMV antigens as the outcome. Column A- difference in level of each CMV antibody between patients and controls and when all other variables are zero. Column B- association of CMV antibody and sBAFF in controls and

(column C) HIV patients. Column D- difference in association of CMV antibody and sBAFF in HIV patients compared to association in controls.
